# Ontogeny of juvenile freshwater pearl mussels, *Margaritifera margaritifera* (Bivalvia: Margaritiferidae)

**DOI:** 10.1371/journal.pone.0193637

**Published:** 2018-03-28

**Authors:** Louise Lavictoire, Andrew D. Ramsey, Evelyn A. Moorkens, Graham Souch, M. Christopher Barnhart

**Affiliations:** 1 Freshwater Biological Association, The Ferry Landing, Far Sawrey, Ambleside, Cumbria, United Kingdom; 2 Centre for Wildlife Conservation, University of Cumbria, Ambleside, Cumbria, United Kingdom; 3 College of Life and Natural Sciences, University of Derby, Derby, United Kingdom; 4 University of Dublin, Trinity College, Dublin, Ireland; 5 Department of Biology, Missouri State University, Springfield, MO, United States of America; Museum National d'Histoire Naturelle, FRANCE

## Abstract

The gills of juvenile freshwater bivalves undergo a complex morphogenesis that may correlate with changes in feeding ecology, but ontogenic studies on juvenile mussels are rare. Scanning electron microscopy was used to examine the ultrastructure and ontogeny of 117 juvenile freshwater pearl mussels (*Margaritifera margaritifera*) ranging in age from 1–44 months and length from 0.49–8.90 mm. Three stages of gill development are described. In Stage 1 (5–9 inner demibranch filaments), only unreflected inner demibranch filaments were present. In Stage 2 (9–17 inner demibranch filaments), inner demibranch filaments began to reflect when shell length exceeded 1.13 mm, at 13–16 months old. Reflection began in medial filaments and then proceeded anterior and posterior. In Stage 3 (28–94 inner demibranch filaments), outer demibranch filaments began developing at shell length > 3.1 mm and about 34 months of age. The oral groove on the inner demibranch was first observed in 34 month old specimens > 2.66 mm but was never observed on the outer demibranch. Shell length (R^2^ = 0.99) was a better predictor of developmental stage compared to age (R^2^ = 0.84). The full suite of gill ciliation was present on filaments in all stages. Interfilamentary distance averaged 31.3 μm and did not change with age (4–44 months) or with size (0.75–8.9 mm). Distance between laterofrontal cirri couplets averaged 1.54 μm and did not change significantly with size or age. Labial palp primordia were present in even the youngest individuals but ciliature became more diverse in more developed individuals. Information presented here is valuable to captive rearing programmes as it provides insight in to when juveniles may be particularly vulnerable to stressors due to specific ontogenic changes. The data are compared with two other recent studies of *Margaritifera* development.

## Introduction

Research and efforts to conserve freshwater mussels (Unionida) increased dramatically during the 20^th^ Century in response to population losses and extinctions [1,2]. A species of particular concern is the freshwater pearl mussel (*Margaritifera margaritifera*) which is critically endangered [3] and has thus been the focus of captive rearing activities in Europe for over 30 years [4–9]. The life history of *M*. *margaritifera* is well documented [10–13]. Parasitic glochidia larvae are released in summer and attach to the gills of salmonid host fish, where they remain over winter and metamorphose to the juvenile stage. The metamorphosed juveniles leave the host (excyst) the following spring and occupy interstitial spaces in the river substrate. Population declines have been attributed particularly to degradation of this interstitial habitat through siltation and pollution [14–16].

As post-parasitic *M*. *margaritifera* grow from about 0.4 mm to several mm in length, the gills (ctenidia) develop from a few simple ciliated filaments to folded structures that transport water through internal spaces [17–19]. It has been suggested that the simple gills of early juvenile mussels must be ineffective for suspension feeding, and that most particle capture instead involves the ciliated foot (pedal feeding [20–25]). If a functional transition occurs as the gills develop, this transition could have implications for feeding ecology and captive culture methods [17,18]. However, the mechanisms involved in food capture by early juveniles are not well documented or understood. Direct internal observation and video imaging of particle capture by Unionids has been accomplished only for adults with fully formed gills [26,27].

**Fig 1:**
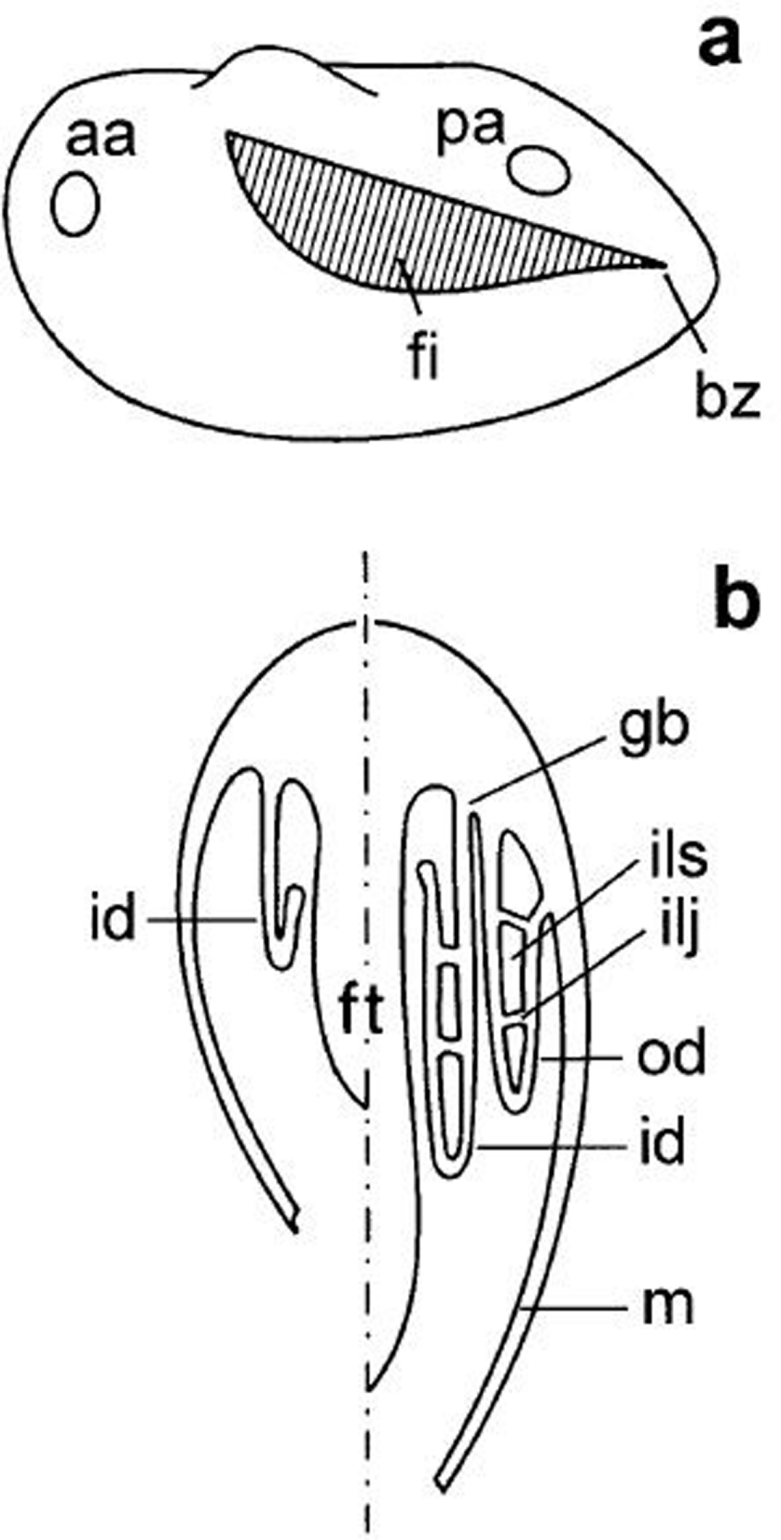
Diagram of gill position and cross-section through a lamella showing ascending and descending limbs of a filament. A: Gill position within a valve showing positioning of the anterior adductor (aa) and posterior adductor muscles (pa), budding zone (bz) and filaments (fi). B: A dorso-ventral section though a eulamellibranch showing the foot (ft), gill base (gb), inner demibranch (id), interlamellar junction (ilj), interlamellar space (ils), mantle (m) and outer demibranch (od). Used with permission from [38].

For reference, the adult structures and terminology are provided in Fig 1. *M*. *margaritifera* is a eulamellibranch mussel displaying the homorhabdic gill condition. Each gill (ctenidium) consists of an inner and outer demibranch. The demibranchs are formed by rows of filaments that descend from the dorsal axis, and reflex upward to define an internal (suprabranchial) space. Each filament bears three sets of ciliary structures. Lateral cilia are responsible for water movement between the filaments, from the branchial (mantle) cavity to the suprabranchial cavity. Laterofrontal cirri are believed to be responsible for particle capture, although debate remains as to whether they act as ‘bats’ or ‘sieves’ moving particles to the frontal cilia [28–32]. The frontal cilia transport particles ventrally towards the oral groove (ventral edge of the folded demibranch), in which particles are transported by cilia anteriorly to the labial palps for sorting into particles which are either transported to the mouth and consumed or egested as pseudofaeces.

Juvenile mussels may have different biological and environmental requirements depending upon their mode of feeding [33] and mortality may increase when developmental changes occur [34–36] due to inability to meet energetic demands during morphogenesis [37].

The aim of the present study was to improve the understanding of the morphological development of the gills of juvenile *M*. *margaritifera* and important additional features, such as the foot, labial palps, siphons, mouth and mantle, under a natural temperature and diet regime. This is important because it allows a better insight into potential high-risk periods for juveniles under natural conditions. The biological structure and ontogeny of juvenile gills and other pertinent structures at different ages were investigated using scanning electron microscopy (SEM). The study utilised captive-cultured individuals of known ages.

## Materials and method

All experimental individuals were cultured at the Freshwater Biological Association, Windermere, UK and originated from a single mussel population (location details can be obtained from the author). Permission for use of juvenile *Margaritifera margaritifera* in this investigation was granted by Natural England and ethical approval was granted by the University of Cumbria. All work was carried out in the laboratories at the Freshwater Biological Association, Windermere, UK, and as such, no permission was required for use of land. Propagation methods are outlined in project reports [39,40] and in [5] but are briefly described here. Water is sourced from Windermere, a mesotrophic lake in Cumbria. Water is filtered to removed particles >20 μm for broodstock adults or 15 μm for juvenile mussels and delivered to the systems as outlined in the reports above. Juvenile mussels were collected in 200 μm plankton nets as they excysted from salmon in 2012, 2014 and 2015 and were transferred into substrate measuring 1–2 mm in either the tray system (2014 and 2015 juveniles) described in [40] or the aquarium system (2012 juveniles) outlined in [5]. Juveniles were maintained in these systems under a natural temperature regime until they were required. Experimental work took place between May 2015 and March 2016. Individuals were selected from three cohorts:

1. 1–8 months old (excysted summer 2015).
2. 13–20 months old (excysted summer 2014).
3. 34–44 months old (excysted summer 2012).

Selection was made by disturbing sediment in the tray system described in [5] and siphoning water through a 0.3 mm sieve to retain mussels. Each individual was transferred to a 10 ml glass tube containing 1 mg ml^-1^ MS-222 [41] to induce the valves to gape and to expose soft tissues. Juveniles were processed with either both valves intact or one valve was removed completely to better observe the structures facing the shell. Juveniles were fixed in 2% glutaraldehyde in 0.1M Sorenson’s Phosphate Buffer (SPB) for <24 hours. They were then washed in SPB (x2) before being dehydrated through 25%, 50%, 75% and 2 x 100% ethanol washes. Two washes (30 minutes) of Hexamethyldisilazane (HMDS) were carried out in place of critical point drying. Samples were mounted onto SEM stubs and sputter coated with gold before being loaded into a Zeiss Leo 1450VP scanning electron microscope. The distance between filaments (interfilamentary space or distance) and distance between laterofrontal cirri couplets were measured from micrographs. Where pictures contained inter-filamentary junctions measurements were taken in the vicinity of junctions because, at this point, interfilamentary space is less variable. Effects of potential tissue shrinkage during sample preparation [42,43] were not quantified. All measurements of features on scanning electron micrographs were taken with ImageJ (version 1.48).

### Data analysis

SPSS (v. 22) was used for data analysis. All data were tested for normality (Shapiro-Wilk) before performing parametric tests. ANOVA’s with *post hoc* Tukey’s HSD tests were performed to test for difference in interfilamentary space among individuals of different ages and to test the number of cilia per laterofrontal cirrus. Linear regression was performed for interfilamentary space against shell length and also for the number of inner demibranch ‘vs’ outer demibranch filaments on individuals where the outer demibranch was present.

## Results

Table 1 outlines summary information of specimens considered for this study. Three stages of juvenile development were observed. The age of specimens is provided in the top right of each micrograph. For some individuals, poor specimen quality meant that high quality micrographs were not always possible. In these cases, micrographs from a different cohort may be used to depict key features; where applicable, this has been outlined in the text.

**Table 1:** Summary information about the ranges of shell length (mm) and number of inner demibranch (ID) filaments for the different age cohorts. The number of specimens considered is also provided (n).

Developmental stage	Age (months)	Shell length (mm)	Number of ID filaments	N
1	1	0.49–0.66	5–6	6
	2	0.58–0.83	Missing data	5
	3	0.65–0.81	6–7	6
	4	0.75–0.94	6–11	6
	8	0.90–1.01	7–9	4
	10	0.47–0.86	4–7	4
2	13	0.81–1.37	6–14	6
	14	0.96–1.28	9–13	5
	15	1.00–1.30	7–12	4
	16	1.13–1.52	9–17	5
	20	1.44–1.45	12–16	2
3	34	2.66–5.90	28–62	6
	44	3.35–8.90	34–94	4

### Gills and labial palp morphogenesis

**Fig 2:**
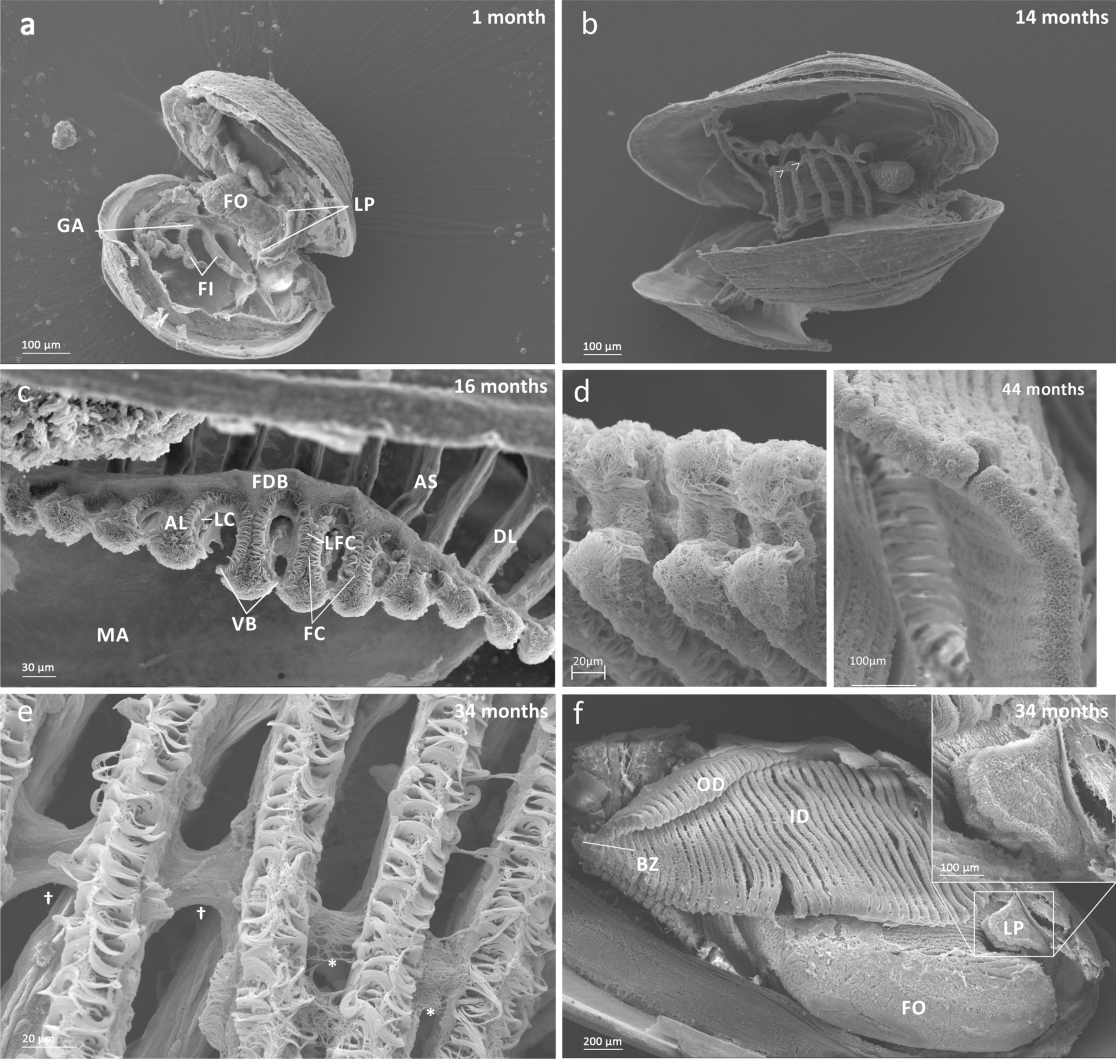
Main anatomical features of juvenile mussels. a) Foot (FO), unreflected filaments (FI), gill axis (GA), left and right labial palps (LP). b) Distal tips of filaments are joined to each other by thin tissue connections (arrow heads); c) Gill reflection of the inner demibranch. Thin tissue connections join filaments at the ventral bend (VB) and the thicker fused dorsal bend (FDB) joins the terminal ends of the ascending arms. All three cilia types are present on the ascending limbs (AL); lateral cilia (LC), laterofrontal cirri (LFC) and frontal cilia (FC). The ascending limb is longer on medial filaments compared to those at either anterior or posterior ends (to the left and right of frame). Other features of note are the filament abfrontal surface (AS), descending limb (DL) and mantle (MA); d) Oral groove on inner demibranch (left) and absence of groove on outer demibranch (right); e) Ciliary junctions between approximately filaments 11–14 (*), after which tissue junctions were present (†); f) Right ID, OD and labial palps (LP). Inset: Labial palps are highly ciliated on the inner surface but devoid of cilia externally.

**Fig 3:**
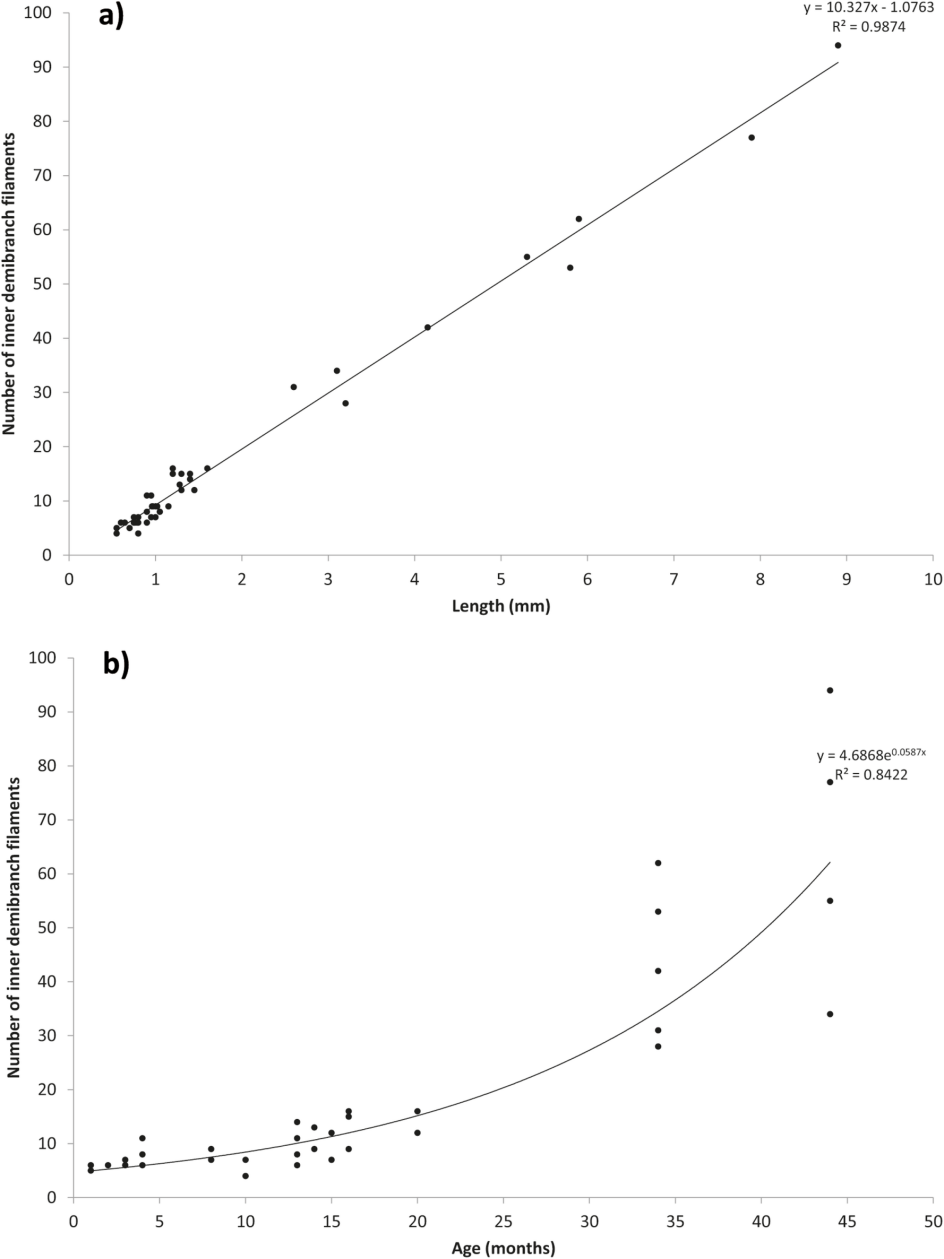
Scatter plots of the number of inner demibranch filaments against shell length (a) and age (b). R^2^ values show shell length is a better predictor of number of inner demibranch filaments compared to age.

The ctenidia of 1 month old individuals consisted of only the inner demibranchs, each consisting of 3–4 simple filaments (Fig 2A). As the mussels grew, new filaments were added to the inner demibranch at the posterior budding zone. The number of filaments increased with shell length, with about 10.3 filaments added per mm shell length over the range of 1.0–8.9 mm (Table 1, Fig 3). Across all specimens, filament diameter averaged 28 μm (± 5 μm) and interfilamentary space 31 μm (± 10 μm). Filaments of 1 month old individuals were unconnected laterally to one another (Fig 2A). Attachment between the tips of adjacent filaments was observed at 3–4 months (> 0.8 mm; Fig 2B of a 14 month old individual). This area of attachment is called the ventral bend. Connections usually began posteriorly near the budding zone and the tissue was covered in simple cilia resembling frontal cilia (see below).

Inner demibranch filaments were added posteriorly and they began to reflect when individuals exceeded about 1.1 mm in length and when there were more than 9 filaments (Fig 2C). Reflection appeared to begin in the middle of the demibranch where the filaments were longer, so that the ascending limbs on the medial filaments were longer than on those and anterior and posterior (Fig 2C). In juveniles older than 34 months, new inner demibranch filaments were budding via cavitation extension. All filaments were reflected except the most anterior filament, which consisted of only a descending limb which was attached to the visceral mass along its entire length. In addition to connection at the ventral bend, filaments were also joined at the terminal end of the ascending limb by a continuous, thick piece of tissue, called the fused dorsal bend (Fig 2C). The fused dorsal bend was never observed attached to the visceral mass, even in the oldest/largest individuals.

No oral groove was observed on the inner demibranch at the size and age when gill reflection commenced (1.13–1.45 mm and 13–20 months old). However, the ventral bend appeared bulbous, which may be a precursor to oral groove development. The oral groove was present on the inner demibranch in older/larger specimens (>34 months and >2.6 mm). The groove was a deep, circular invagination on filaments that appeared anteriorly of approximately filaments 2–13 (numbering from posterior–anterior; Fig 2D).

Interfilamentary junctions were not observed in younger/smaller individuals but were observed from 34 months (2.66 mm). Filaments 1–11 had no interfilamentary junctions, filaments 11–15 had ciliary junctions, and more anterior filaments had tissue junctions (Fig 2E). Longer, more anterior filaments also exhibited additional interfilamentary junctions along their dorso-ventral length. In the largest individual considered during this study (shell length = 8.9 mm) the inner demibranch had 6 rows of tissue interfilamentary junctions in a dorsal-ventral direction. No ciliary interfilamentary junctions were observed in this individual.

Filaments of the outer demibranch first appeared in individuals larger than 3.1 mm. Outer demibranch filaments proliferated via cavitation extension (Fig 2F), giving rise to several filaments at once. The ventral bend on the outer demibranch was covered in simple cilia, and was flattened but not invaginated into an oral groove as observed on the inner demibranch (Fig 2F).

**Fig 4:**
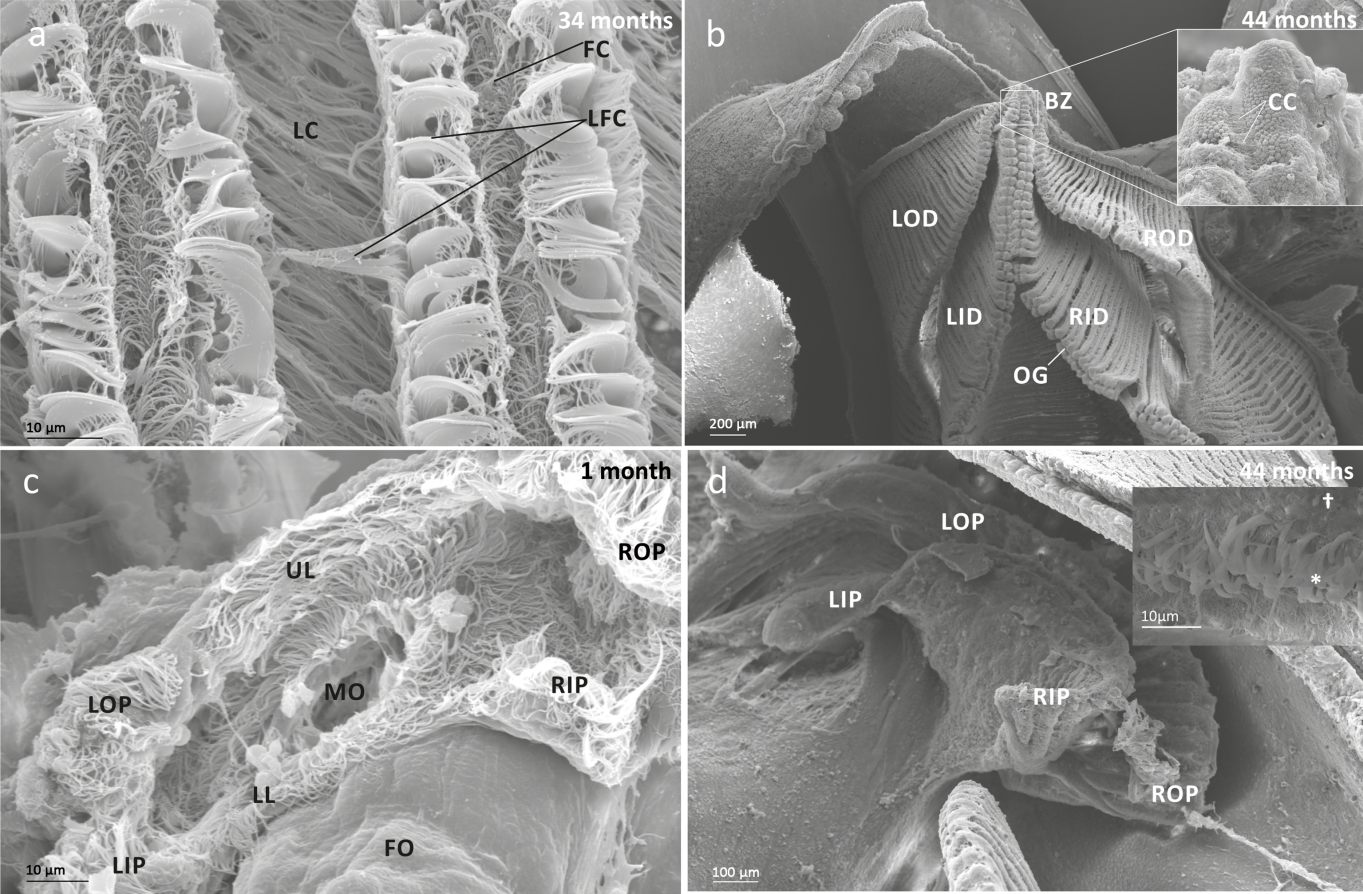
Main anatomical features of juvenile mussels. a) Ciliation of gill filaments; frontal cilia (FC), lateral cilia (LC) and laterofrontal cirri (LFC); b) Budding zone (BZ) and left inner (LID), left outer (LOD), right inner (RID) and right outer demibranchs (ROD). Inset box shows ciliary connection (CC) between left and right BZ. OG = oral groove; c) Labial palp primordia, lips and mouth; Foot (FO), left inner palp (LIP), left outer palp (LOP), lower lip (LL), mouth (MO), right inner palp (RIP), right outer palp (ROP), upper lip (UL); d) The labial palps were plicated internally but retained a flattened appearance on the outer surface. Inset shows simple cilia (†) and more complex cirri (*).

The full complement of filament ciliature (frontal and lateral cilia and laterofrontal cirri) was observed on the inner demibranch filaments in all age classes examined (Fig 4A). Laterofrontal cirri were complex, branching and had cirral plates orientated perpendicular to the filament. Each laterofrontal cirrus consisted of two parallel rows of cilia which were shortest towards the frontal surface and became progressively longer towards the lateral surface. Ciliation on the developing ascending limb was the same as on the descending limb with all types of cilia/cirri present although laterofrontal cirri were smaller and consisted of fewer cilia per cirrus. Laterofrontal cirri could reach over half way across interfilamentary spaces only in more developed individuals (as shown in Fig 4A). In all age groups, the abfrontal surface of filaments had only a very sparse, unorganised coverage of simple cilia.

The filament budding zone was not very prominent in smaller individuals but in larger specimens it was covered in simple cilia. Gill buds were distinguished from ‘true’ filaments by the absence of laterofrontal cirri. Ciliary connections joined the left and right parts of the budding zone (Fig 4B). Each individual had approximately 3–5 buds before the onset of true filaments and the budding zone was not connected to the mantle. Laterofrontal cirri on newly-budded filaments consisted of fewer cilia per cirral plate (i.e. not as wide) as laterofrontal cirri on older filaments.

On younger individuals, labial palp primordia were observed as paired projections on either side of the mouth and were densely covered with simple cilia (Fig 4C). By 20 months old (~1.45 mm long), the labial palps were becoming plicate and a ciliary connection was also observed between the labial palp and the penultimate anterior filament in one individual. In older individuals the labial palps were plicate with both simple cilia and more complex cirri present on the inner but not the outer surfaces (Fig 4D).

### Gill morphometry

**Fig 5:**
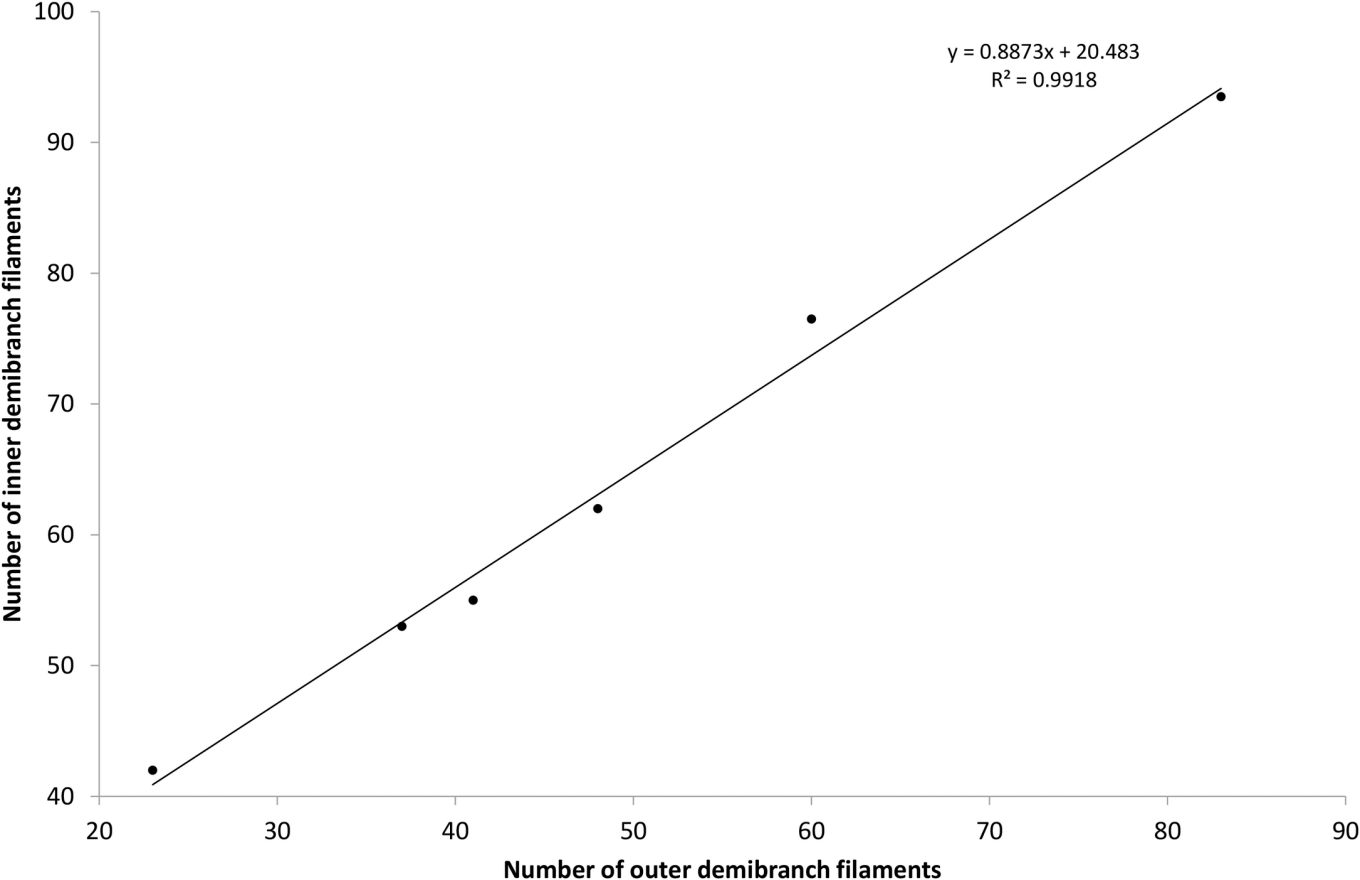
Scatter plot of the number of inner ‘vs’ outer demibranch filaments. The number of inner demibranch filaments is able to predict the number of outer demibranch filaments and accounted for 99% of the explained variability in number of outer demibranch filaments.

Juvenile shell length was closely correlated with the number of inner demibranch filaments (n = 47; F_(1,45)_ = 3520; *P* < 0.001; R^2^ = 0.99) and length was a better predictor than age (R^2^ = 0.84; Fig 3). The number of inner demibranch filaments also correlated with the number of outer demibranch filaments (Fig 5; F_(1,4)_ = 483, *P* < 0.001, R^2^ = 0.99).

**Fig 6:**
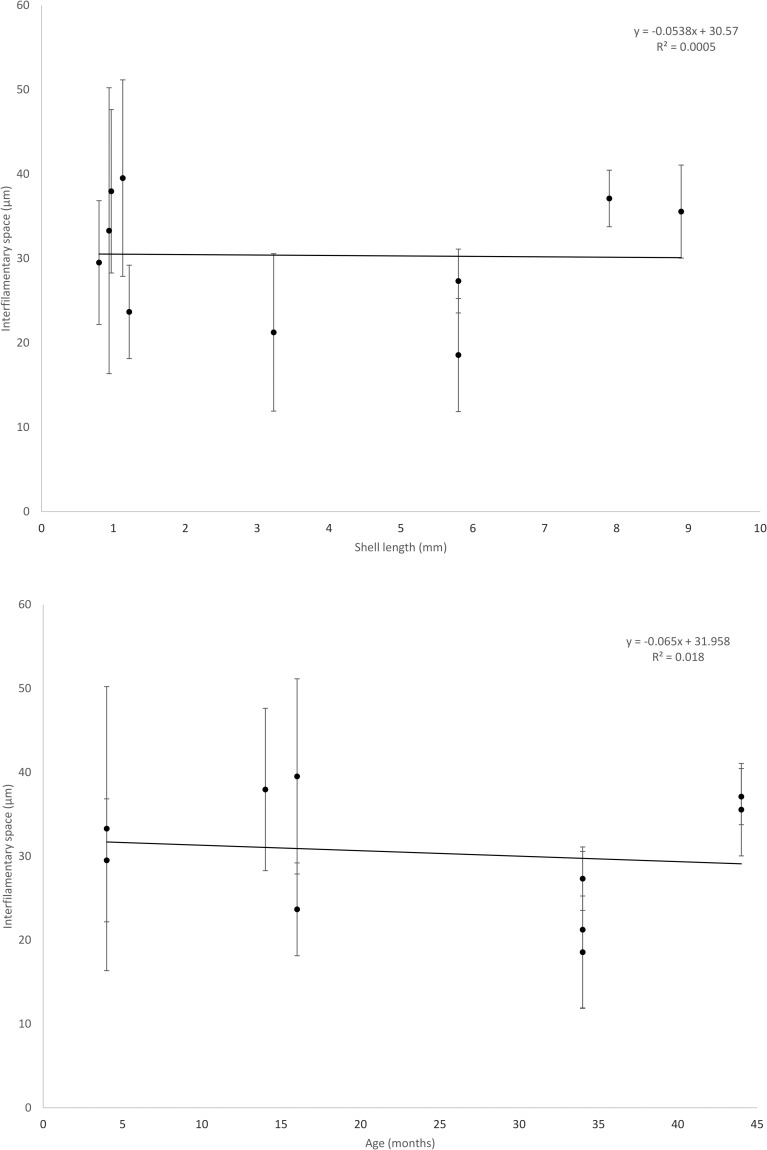
Interfilamentary space vs length (mm) and age (months). There was no significant trend of interfilamentary space with length nor age.

The space between filaments differed significantly among individuals (F_(4,74)_ = 11.40, *P* < 0.001) but did not show any significant trend with size or age (Table 2, Fig 6). The distance between filaments was more consistent when tissue junctions were present compared to ciliary junctions or no junctions (SD of interfilamentary distance was larger in individuals < 16 months old).

**Table 2:** Mean interfilamentary space and filament diameter of individual specimens at given ages and shell lengths. The number of measurements taken from each individual is also provided (n).

Individual identifier code	Length (mm)	Age (months)	Mean interfilamentary space (μm)	Mean filament diameter (μm)	N
1011-15-1	0.94	4	33 (±17)	29 (±2)	3
1011-15-6	0.8	4	30 (±7)	29 (±3)	3
039-14-1	0.97	14	38 (±10)	18 (±3)	4
1011-14-9	1.22	16	24 (±6)	26 (±5)	7
1011-14-10	1.13	16	40 (±12)	25 (±2)	8
185-12-9	5.8	34	27 (±4)	27 (±2)	4
185-12-10	3.23	34	21 (±9)	26 (±4)	10
185-12-9	5.8	34	19 (±7)	31 (±2)	7
103-12-2	7.9	44	37 (±3)	34 (±4)	14
103-12-1	8.9	44	36 (±6)	28 (±3)	18

The number of cilia per laterofrontal cirrus increased as filaments developed with fewer cilia per cirrus on newly budded filaments and also on filaments of the ascending limb in juveniles undergoing gill reflection. However, comparing only cirri on the descending limb of reflecting filaments, there was no difference in the number of cilia per cirrus among 4 month olds (42 ± 2, n = 6), 16 month olds (43 (± 3), n = 3) and 34 month olds (39 (± 9), n = 2). Laterofrontal cirri couplets were spaced an average of 1.54 μm (± 0.40) apart (n = 21 from three 16 & 34 month old individuals).

The processes and timing of gill development were used to categorize three stages of development related to size and age. These stages are outlined in Table 3.

**Table 3:** Description of the three stages of juvenile gill development based upon observations during this study. The age (months) at which individuals begin to display particular structures/developments is approximate and no attempt has been made to postulate when development of certain structures begins if they were not directly observed during this study. The number of inner demibranch (ID) and outer demibranch (OD) filaments are the number observed during this study and may differ depending upon population or other parameters.

Stage	Age (months)	Shell length (mm)	Description	No. of ID filaments	No. of OD filaments
1	0–13	0.40–1.13	Proliferation of unreflected filaments with the gradual formation of connections between adjacent filaments at the ventral bend in individuals ~4 months old (~0.75 mm). Labial palp primordia simple, flat and not plicated but heavily ciliated. No oral groove on inner demibranch.	5–9	0
2	13–20	1.13–1.45	Filaments commence reflection starting with the medial filaments at shell length ~1.2 mm. Ascending limb joined at fused dorsal bend which is covered by simple cilia. Labial palps becoming larger and starting to take on plicated morphology. No oral groove on inner demibranch. No outer demibranch development.	9–17	0
3	34–44	2.66–8.90	Reflected filaments on inner demibranch with new filaments developing via cavitation extension. Budding zone obvious giving rise to 3 - 5 buds before true filaments develop. Oral groove develops after 2–13 true filaments on the inner demibranch. Outer demibranch proliferation via cavitation extension in individuals >3 mm long. First sighting of ciliary and tissue interfilamentary junctions on inner demibranch.	28–94	0–83

### Foot

**Fig 7:**
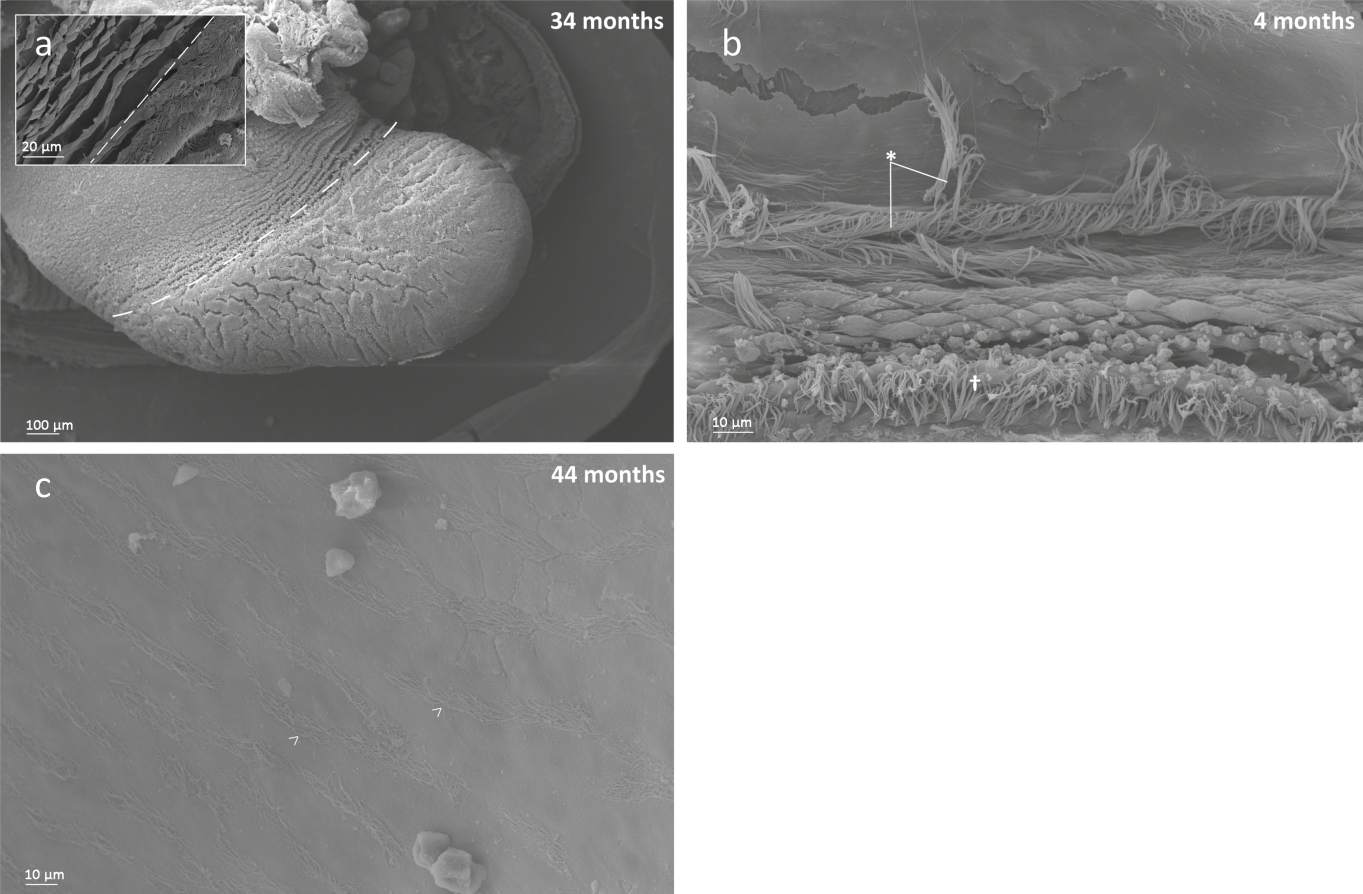
Notable foot and mantle features in juveniles of all age/size classes. a) The foot showing two distinct regions. The distal portion, below and right of the ‘heel’ (dashed line) is heavily ciliated while the proximal region, above and left of the ‘heel’, bears only sparse ciliation. Inset shows ciliation in greater detail; b) Long simple cilia (*) and short cirral tracts (†) near the mantle margin. Ventral shell margin is to the bottom of the image; c) Mantle surface covered in rows of cilia (arrow heads).

Foot form and ciliation was consistent throughout all age classes. The foot appears to have two distinct regions (Fig 7A). The distal region is folded and has a dense coverage of simple cilia whilst the proximal region above the ‘heel’ is only very sparsely covered with patches of cilia. The largest 44 month old juvenile (8.9 mm), had a fine byssus thread attached. A thin hole was observed along the distal tip of the foot which was likely the byssus pit.

### Mantle

In young individuals before ctenidia organogenesis, simple cilia were visible around the mantle margin with more dense aggregations near the posterior end. Ciliation was arranged into three rows. Firstly a band of short cirri composed of 6–10 individual cilia was present closest to the margin with two bands of long simple cilia above this (Fig 7B). Sparse aggregations of long simple cilia were distributed over the visible part of the mantle and occasional instances of long cilia in two parallel rows were observed. Ciliation continued to develop in individuals undergoing ctenidia organogenesis, becoming denser, especially posteriorly around the areas where siphons would eventually develop. By 44 months old the mantle surface was covered in organised rows of simple cilia (Fig 7C) with ciliation becoming denser close to the inhalant siphon. Tissue making up the inhalant siphon was plicated and ciliation extended to the shell-facing side of the mantle.

## Discussion

This paper is among the first attempts [17–19] to describe the ontogeny of gill ultrastructure of juvenile freshwater pearl mussels *Margaritifera margaritifera* with scanning electron microscopy (SEM) and the first to investigate development under a natural temperature regime for this species. Earlier studies of the adult anatomy of freshwater mussels were directed mainly at taxonomic relationships [44–46] and reproduction [47]. It is hoped that understanding juvenile gill development can improve understanding of feeding mechanisms and lead to evidence-based decisions about captive rearing practices. This study collected data from 117 juvenile mussels, focussing mainly on the ontogeny of ctenidia and associated structures. The ability of the ctenidia to efficiently capture particles is thought to depend on small interfilamentary space [48], development of the oral groove for particle transport to the labial palps [49], contact between anterior gill filaments and the labial palps [50] and the ability of ciliary mechanisms to drive a current of water through the mantle cavity to deliver suspended particles.

*Margaritifera margaritifera* undergoes substantial gill ontogeny during the first 44 months post-excystment (up to 8.90 mm). In Stage 1, ctenidia grow via simple proliferation of unreflected filaments until shell length > 0.8 mm (around 3–4 months old) when filaments begin to connect at the distal tips. The laterofrontal cirri are complex, branching and have a similar structure to those observed in other lamellibranch bivalves [32,49,51], but may not form an efficient sieve at this stage because the interfilamentary spaces are too wide. The labial palps at this stage are simple, flattened flaps which are not plicate at this stage but are heavily ciliated, as is the inner surface of the lips and the area around the mouth.

Stage 2 commences with the onset of gill reflection between 13–16 months old when shell length ≈1.2 mm, and when the inner demibranch consists of > 9 filaments. Shell length and the number of inner demibranch filaments attained before the onset of gill reflection is similar to those previously reported in other bivalve species [35,37,50,52]. However, *M*. *margaritifera* takes over a year, under the natural temperature regime in NW England, to commence gill reflection, the most delayed onset reported for any freshwater bivalve [19]. Medial inner demibranch filaments begin to reflect first, followed by anterior and posterior filaments. Once the most posterior filaments have reflected, proliferation from the budding zone is via cavitation extension. The number of inner demibranch filaments added per mm shell length in this study is consistent with results from Schartum et al. [17] (10.33 and 8.10 respectively). With further development during Stage 2, tissue at the ventral bend becomes thicker and more densely ciliated, probably as a precursor to oral groove development [50]. The outer demibranch is not present at this stage.

Stage 3 involves several important morphological changes. Firstly, the outer demibranch develops in individuals > 3.1 mm long and both the inner and outer demibranch filaments proliferate via cavitation extension, the same as reported for *Crassostrea gigas* [36]. The number of filaments on the inner and outer demibranch were highly correlated (R^2^ = 0.99). The age/size at which the outer demibranch develops is most likely species-specific.

Secondly, the oral groove begins to develop on the inner demibranch when individuals reach a shell length of between 1.45–2.60 mm. This result concurs with Araujo *et al*. [18] who found that the oral groove was present in 1.8 mm juveniles. The oral groove in *M*. *margaritifera* remains open, unlike the deep, enclosed oral grove of *M*. *edulis* [53] and is present anterior of approximately the 2 – 13^th^ filament. The oral groove was never observed on the outer demibranch in the size-age range examined. Possibly the particles collected by the outer demibranch are passed to the frontal surface of the inner demibranch for transport to its oral groove [54,55]. Absence of an outer demibranch oral groove appears to be the norm in juvenile bivalves excepting *M*. *edulis* [53].

Thirdly, interfilamentary junctions on the elongating inner demibranch filaments were observed for the first time in Stage 3, beginning as ciliary junctions but quickly giving way to tissue junctions, similar to other eulamellibranchs [36,50,53]. Interfilamentary junctions developed in individuals >2.66 mm long (34 months old), it is likely that their development begins in slightly smaller and younger individuals. No ciliary junctions were observed in 44 month old specimens and it may be that only tissue junctions form after a certain size/age. Where several rows of interfilamentary junctions were present they appeared to be spaced approximately evenly along the dorso-ventral axis, likely to provide stability to elongating filaments. Addition of interfilamentary junctions along the dorso-ventral axis suggests that the site of elongation i.e. ventral growth of filaments, may be from the ventral portion of the filament rather than from the gill axis. Initial growth of the ascending lamella upon reflection is from the ventral bend region [38,53,56] but elongation may be from the terminal end of the ascending lamella i.e. near the fused dorsal bend. This area warrants further investigation. The current study concurs with the sequence of developmental stages [17] reported previously, at least until 44 months of age (shell length = 8.9 mm) (Table 3). Filament diameter (28 ±5 μm) and interfilamentary space (31 ±10 μm) in this study displayed isometry (Fig 6) and these dimensions are similar to those reported by Schartum et al. (28 μm and 29 μm respectively; [17]).

A substantial body of research exists on the topic of gill ciliation, particularly the role of laterofrontal cirri in particle capture [57–61]. In the present study, the full suite of frontal and lateral cilia and laterofrontal cirri were present in even the youngest individuals and filaments developed cilia and cirri almost as soon as they were budded, similar to previous reports for other bivalves, both freshwater [50,52] and marine [36,53]. The structure of laterofrontal cirri in *M*. *margaritifera* is similar to previously described [32,48,51] and their function, whilst not observed directly in this study, is believed to be similar also. That is, cirri from opposing paired plates splay out in turn in opposing directions either acting as ‘nets’ to sieve particles or ‘paddles’ to create vortices that transfer particles to the frontal surfaces of the filaments [32,42,48,51,56,60–62]. Whilst the number of cilia per laterofrontal cirrus is variable between species (S1 Table), the number in *M*. *margaritifera* was comparable to other marine and freshwater bivalve species.

Cirral plates in *M*. *margaritifera* are more closely aligned (1.54 μm ± 0.40) compared to other species (2.0–3.5 μm; [34,45–47]). This type of laterofrontal cirrus is efficient at capturing small particles [63] and close alignment of cirral plates suggests that *M*. *margaritifera* may be capable of retaining particles < 2 μm, even smaller than previously suggested [64]. The relationships between gill structure, feeding mechanisms and ecological niche are important considerations for researchers and practitioners working with endangered mussel conservation and population recovery. If *M*. *margaritifera* is capable of retaining particles <2 μm and is also has a poor ability to select nutritious particles over non-nutritious particles [64], this species may be vulnerable to changes in the size and composition of particles in its interstitial environment. Sustainable recruitment is no longer observed in many populations. Historically oligotrophic conditions no longer exist in the majority of modern pearl mussel rivers. Silt loading in rivers introduces inorganic material not suitable for consumption that must be ejected in pseudofaeces, or lower the nutritional value of ingested material. Nutrient loading stimulates unnaturally high concentrations of relatively large algal cells, some of which may not be in the correct size range for mussels to consume. This again has energetic consequences of either egestion as pseudofaeces or restricting filtering to avoid the particles [65–67]. Propagation facilities providing supplementary feeding to juvenile mussels might consider the implications of gill ciliation on the ability of juveniles to feed on the particles provided. Inter-cirral distance has been shown to increase with age in the marine bivalve *Perna canaliculus* [48] which may alter food particle size preference with increasing age. This was not observed in *M*. *margaritifera* in the size and age cohorts studied.

This study also made observations of other structures important for juvenile feeding. The labial palps developed significantly throughout this investigation. Labial palps began as small simple structures which grew and became plicate at approximately 20 months old (shell length = 1.45 mm). It is possible that this marks the beginning of more complex sorting capabilities of the labial palps although this hypothesis needs to be tested with further observations.

Foot ciliation showed a consistent pattern in all age classes studied. The distal portion was heavily invested with short simple cilia. These cilia are very active during locomotion and could resuspend deposited particles that then would be drawn into the pedal gape. This pattern of ciliation is similar to previous descriptions in other species [50,60,68] and supports the hypothesis that pedal cilia, along with mantle and gill cilia, have a function in feeding. However, there was no evidence of pedal ciliary tracts for direct particle transport.

At all developmental stages, mantle ciliation was denser around the posterior region where the apertures eventually develop. Ciliation was also present around the majority of the mantle margin, where cilia were arranged into areas of short, compound cirri nearest the margin and longer, simple cilia dorsal to this. Longer cilia may have a role in pseudofaeces transport [43]. The shorter cirri may have a role in creating water currents but direct observation in live specimens is required to confirm this. Ciliation elsewhere on the mantle was unorganised and patchily distributed in younger individuals, becoming more organised into tracts by 44 months old. It is possible that these cilia are involved in maintaining water currents through the branchial cavity. By 44 months old siphons were well-developed and the inhalant siphon had the characteristic papillose form and was highly ciliated.

Attainment of true siphonal filter feeding is likely to be a gradual process with individuals probably exploiting both pedal and siphonal filtering mechanisms concurrently for some time. In small juveniles, the entry of water into the mantle cavity is not limited to the posterior and also occurs along the anterior and ventral margins [20]. Limitation of water entry to the posterior incurrent aperture is likely to be a gradual process that culminates when individuals achieve a size and flow rate that requires access to the water column rather than the interstitial space.

### Conclusions and implications for captive rearing programmes

Proliferation of freshwater mussel propagation programmes within at least the last 30 years [2,6,69] has necessitated increased understanding of the factors affecting juvenile growth and survival in captivity. Ontogenic studies are important to complement ecological and genetic studies and help improve understanding of endangered species in order to develop comprehensive management plans [70,71].

This work details information valuable to captive rearing programmes regarding the timing of key developments, how this may affect the survival and adaptation of *M*. *margaritifera* to oligotrophic conditions. A summary of key biological developments over the first four years post-excystment is provided in Table 4 including details of factors potentially important to juvenile survival in captivity, as well as providing insight into the most sensitive periods where pressures may impact efficient feeding and development in the wild. The current situation of enriched habitat conditions with unnaturally high suspended solids concentrations are assumed for this table.

**Table 4:** Summary of main periods during the first approximately 4 years post-excystment outlining whether the mortality risk is deemed to be low, medium or high and which factors may contribute to increased mortality during those periods. Possible mitigation measures are also provided.

Timing	Age (months)	Mortality risk	Important considerations and potential risk factors	Mitigation
First growth season (June–October)	0–4	High	• Insufficient reserves laid down as glochidia. • Minimally-effective filter-pump so must be very active to forage—high metabolism and DO requirement. • Higher summer temperatures leading to: ∘ Lower DO concentrations. ∘ Higher primary production of potentially unsuitable algal species leading to decreased filtration rate (clamming).	• Select only the most active juveniles for culture • Ensure high DO concentrations at all times • Filter water supply to remove unsuitable organic and inorganic particles
First winter (October–May)	4–11	Low	• No significant ontogenic changes. • Low winter temperatures so low DO risk factors. • Primary production lower than in summer so less energy required to clear excessive/unsuitable algal loads. • Potential for higher suspended solids concentrations during winter flood conditions.	• Filter water supply to avoid delivery of fine suspended sediment to juveniles
Second growth season(May–October)	11–16	High	• Gill reflection begins (size dependent)–may require excess energy/increased metabolism*. • Higher summer temperatures leading to: ∘ Lower DO concentrations. ∘ Higher primary production of potentially unsuitable algal species leading to decreased filtration rate (clamming).	• If providing supplementary feeding, concentrations may need to be increased* • Ensure high DO concentrations at all times • Filter water supply to remove unsuitable organic and inorganic particles
Second winter (October–May)	16–23	High	• Gill reflection begins/is ongoing (size dependent). May require excess energy/increased metabolism*. • Potential for higher suspended solids concentrations during winter flood conditions.	• If providing supplementary feeding, concentrations may need to be increased* • Filter water supply to avoid delivery of fine suspended sediment to juveniles
Third growth season (May–October)	23–28	Medium/High	• Development of oral groove and outer demibranch begins. Possible other ontogenic changes during this period–requires further investigation. • Higher summer temperatures leading to: ∘ Lower DO concentrations. ∘ Higher primary production of potentially unsuitable algal species leading to decreased filtration rate (clamming).	• If providing supplementary feeding, concentrations may need to be increased* • Ensure high DO concentrations at all times • Filter water supply to remove unsuitable organic and inorganic particles
Third winter (October–May)	28–35	Low	• Outer demibranch development progresses (size dependent). May require excess energy/increased metabolism*. • Potential for higher suspended solids concentrations during winter flood conditions.	• If providing supplementary feeding, concentrations may need to be increased* • Filter water supply to avoid delivery of fine suspended sediment to juveniles
Fourth growth season(May–October)	35–40	Medium	• Higher summer temperatures leading to: ∘ Lower DO concentrations. ∘ Higher primary production of potentially unsuitable algal species leading to decreased filtration rate (clamming).	• If providing supplementary feeding, concentrations may need to be increased* • Ensure high DO concentrations at all times • Filter water supply to remove unsuitable organic and inorganic particles
Fourth winter season(October–May)	40–47	Low	• Unknown biological development–requires further investigation. • Potential for higher suspended solids concentrations during winter flood conditions.	• Filter water supply to avoid delivery of fine suspended sediment to juveniles

*N*.*B*. *The mortality risk depends upon juvenile size and developmental stage and may therefore occur at different times in other captive rearing systems*. *Practitioners should consider how their individual culture systems may affect juvenile development and may therefore affect the timing of high-risk periods*.

*N*.*B*. * Not considered as part of this study. *Requires further investigation*.

Gill reflection begins to occur around the middle/end of the second growth season (shell length >1.20 mm; 13-16 months old). Gill folding has the effect of increasing the surface area of the ctenidia relative to the dimensions of the mantle cavity. The effect of gill folding, in terms of the rates at which water transport and filtration occur, has not yet been directly tested. It would be useful and interesting to extend measurement of the allometric relationship between mass-specific filtration rate and size [61] into the size range of this developmental transition. Another area ripe for investigation is the relationship between filtration rate, interstitial space, and hydraulic conductivity of sediments, which together may determine when juvenile *M*. *margaritifera* must migrate to the substrate surface.

Whilst this investigation considered juveniles from only one population of *M*. *margaritifera*, some important conclusions can be drawn about how juvenile ontogeny may affect survival in captivity:

1. Reported mortality during the first few months post-excystment [5,22,72] is not related to ctenidia organogenesis. This high mortality rate does not correlate with substantial ontogenic changes in gill morphology. Insufficient availability of appropriate food particles or feeding avoidance behaviour/over-production of pseudofaeces due to turbid habitat conditions may result in a net loss of energy, leading to mortality. Future work should focus on initial juvenile quality and optimising environmental conditions for the youngest/most vulnerable juveniles.
2. Juveniles may be particularly sensitive to stress factors when gill reflection commences. Additional somatic reserves may be required to undergo significant morphological changes and the timing of reflection suggests that juveniles may have to store additional nutritional reserves over the second growth season to meet this proposed increased demand. Therefore, stress factors (Table 4) should be kept to a minimum during the second growth season and second winter. Future studies should consider the abundance of compounds such as lipids and polysaccharides for juvenile growth at all stages of development.
3. The complex nature of laterofrontal cirri, the high numbers of cilia per laterofrontal cirrus and small inter-cirral distance implies that juvenile *M*. *margaritifera* are capable of filtering particles < 2 μm. This may limit the ecological niche of *M*. *margaritifera* to oligotrophic streams and may partially explain why recruitment has stalled in most surviving populations. This hypothesis is supported by the findings of ecological studies on the loss of juvenile function with increased catchment intensification and loss of oligotrophic conditions [73, 74].

These observations and initial measurements have increased our understanding of early *Margaritifera* development. Ontogenic studies are important to complement ecological and genetic studies and help improve understanding of endangered species such as the freshwater pearl mussel in order to develop comprehensive management plans.

## Supporting information

S1 Table Number of cilia per laterofrontal cirrus in various lentic and lotic freshwater and marine bivalves. (DOCX)
