# Supplementary material for: Ontogeny of juvenile freshwater pearl mussels, Margaritifera margaritifera (Bivalvia: Margaritiferidae)
Source: PLoS One. 2018 Mar 28;13(3):e0193637. doi: 10.1371/journal.pone.0193637 (PMC5873991; doi:10.1371/journal.pone.0193637)
Supplement: S1 Table — (DOCX) [file pone.0193637.s001.docx]

**S1 Table: Number of cilia per laterofrontal cirrus in various lentic and lotic freshwater and marine bivalves.**

| Species | Number of cilia/laterofrontal cirrus | Lentic/lotic/marine species? | Reference |
| --- | --- | --- | --- |
| *Margaritifera margaritifera* | 32 – 46 | Lotic | This study |
| *Dreissena polymorpha* | 38 – 42 | Lentic & lotic | [50] |
| *Mytilus edulis* | 22 – 26 | Marine | [54] |
| *Utterbackia imbecilis*  *Toxolasma texasensis*  *Ligumia subrostrata*  *Elliptio dilatata*  *Lampsilis ovata*  *Ptychobranchus fasciolaris*  *Fusconaia flava*  *Villosa lienosa*  *Cyclonaias tuberculate* | 14 – 15  11 – 13  12 – 16  30 – 32  26 – 35  25 – 32  25 – 30  25 – 30  38 – 42 | Lentic  Lentic  Lentic  Lotic  Lotic  Lotic  Lotic  Lotic  Lotic | [55] |
| *Actinonaias ligamentina Amblema plicata Obliquaria reflexa Fusconaia flava* | 13 – 23 6 – 16  21 – 26  15 – 21 | Lotic  Lentic & lotic  Lotic  Lotic | [51] |
| *Carunculina texasensis* | 11 – 13 | Lotic | [52] |
| *Perna canaliculus* | 20 – 28 | Marine | [18] |
